# The Association between Salivary Metabolites and Gingival Bleeding Score in Healthy Subjects: A Pilot Study

**DOI:** 10.3390/ijms25105448

**Published:** 2024-05-17

**Authors:** Rita Antonelli, Elena Ferrari, Mariana Gallo, Tecla Ciociola, Elena Calciolari, Alberto Spisni, Marco Meleti, Thelma A. Pertinhez

**Affiliations:** 1 Department of Medicine and Surgery, Centro Universitario di Odontoiatria, University of Parma, 43126 Parma, Italy; rita.antonelli@unipr.it (R.A.); elena.calciolari@unipr.it (E.C.); marco.meleti@unipr.it (M.M.); 2 Laboratory of Biochemistry and Metabolomics, Department of Medicine and Surgery, University of Parma, 43125 Parma, Italy; elena.ferrari@unipr.it (E.F.); mariana.gallo@unipr.it (M.G.); thelma.deaguiarpertinhez@unipr.it (T.A.P.); 3 Laboratory of Microbiology and Virology, Department of Medicine and Surgery, University of Parma, 43125 Parma, Italy; tecla.ciociola@unipr.it; 4 Center for Oral Clinical Research, Institute of Dentistry, Barts and The London School of Medicine and Dentistry, Queen Mary University of London, London E1 4NS, UK

**Keywords:** saliva, Full-Mouth Bleeding Score (FMBS), gingivitis, salivary metabolomics, salivary diagnostics

## Abstract

Periodontal diseases, including gingivitis and periodontitis, are among the most prevalent diseases in humans. Gingivitis is the mildest form of periodontal disease, characterized by inflammation of the gingiva caused by the accumulation of dental plaque. Salivary diagnostics are becoming increasingly popular due to the variation in saliva composition in response to pathological processes. We used a metabolomics approach to investigate whether a specific saliva metabolic composition could indicate preclinical stage of gingivitis. ^1^H-NMR spectroscopy was used to obtain the salivary metabolite profiles of 20 healthy subjects. Univariate/multivariate statistical analysis evaluated the whole saliva metabolite composition, and the Full-Mouth Bleeding Score (FMBS) was employed as a classification parameter. Identifying a signature of specific salivary metabolites could distinguish the subjects with high FMBS scores but still within the normal range. This set of metabolites may be due to the enzymatic activities of oral bacteria and be associated with the early stages of gingival inflammation. Although this analysis is to be considered exploratory, it seems feasible to establish an FMBS threshold that distinguishes between the absence and presence of early inflammatory alterations at the salivary level.

## 1. Introduction

Periodontal diseases affect most of the adult population [1]. Particularly, severe periodontitis, a major irreversible cause of tooth loss, has a prevalence of 11% and is the sixth most prevalent disease worldwide [2,3,4]. Remarkably, periodontitis has been correlated with several inflammatory-based systemic diseases, such as diabetes, cardiovascular diseases, rheumatoid arthritis, Alzheimer’s, and pulmonary infections, as well as adverse pregnancy outcomes [4,5,6,7,8,9]. Moreover, it affects vulnerable segments of the population, negatively impacts quality of life, and is a consequence of social inequality [10].

Plaque-induced gingivitis is a site-specific inflammatory condition caused by microbial plaque accumulation in the gingival sulcus [11,12,13]. Such inflammation remains confined to the gingiva and is reversible by reducing plaque with professional dental cleaning and practicing good oral hygiene (brushing and flossing) [14]. In addition to pathogenetic microorganisms and the host immune response, genetic and environmental factors (e.g., tobacco use and plaque retentive factors) contribute to its development [15]. Clinically, gingivitis affects the marginal gingiva, causing erythema, edema, bleeding on probing, and sometimes increasing gingival volume [11]. If not treated, gingivitis can eventually lead to periodontitis in susceptible subjects [16]. Yet, early diagnosis of gingivitis and periodontitis can be challenging due to their slow progression.

Overall, the presence, extent, or severity of bleeding is the most widely accepted indicator of the prevalence of gingivitis, and according to the current classification of periodontal and peri-implant diseases, bleeding on probing is the primary sign for the diagnosis of gingivitis [12]. The Full-Mouth Bleeding Score (FMBS), which is the percentage of sites in the entire dentition that bleed on probing, can provide an overall assessment of gingival inflammation; a patient with an intact periodontium is diagnosed as a case of gingivitis according to an FMBS ≥10%. In turn, an FMBS < 10% excludes gingivitis and is generally consistent with the patient’s perception of healthy gums [13].

The diagnostic approach to gingivitis and periodontal disease could be revolutionized by detecting salivary components associated with the early development of gingival changes, even at a subclinical level, before bleeding on probing manifests.

Whole saliva, a mixture of fluids produced by the major and minor salivary glands and crevicular fluid, includes endogenous molecules, components derived from the oral microflora, and exogenous substances [17]. Its composition, the result of a dynamic exchange between microflora and mucosa cells, as well as components derived from capillary blood, holds significant clinical relevance. For this reason, saliva is an excellent tool for the identification of clinically relevant biomarkers [18,19,20,21]. Reliable biomarkers specific to a disease can provide useful information regarding the type, molecular etiology, and stage of the disease, driving the development of personalized therapeutic interventions [22,23]. The advantages of using saliva for disease diagnosis include ease of access, non-invasive sample collection, increased patient acceptance, and reduced risk of infectious disease transmission. 

Biofluid-based metabolomics has generated vast knowledge over the past decades. In the case of saliva, research studies have focused on the characterization of the salivary metabolome in relation to oral function, oral microbiome, and the identification of disease biomarkers [19,24,25]. Recent literature reviews have reported using salivary metabolomics to diagnose systemic diseases, systemic cancers, and mental illnesses [25,26]. Regarding the oral cavity, many publications on salivary metabolomics based on whole saliva have focused on the discovery of diagnostic biomarkers for oral cancer [27,28] and periodontitis [29,30]. NMR-based metabolomics provides detailed, qualitative, and quantitative information that is valuable for discovering specific biomarkers. Its routine use for screening or diagnostic purposes is limited due to the hardware and maintenance costs. Nevertheless, once a biomarker (or a pattern of biomarkers) has been identified, it can be employed to develop novel, reliable, and non-invasive devices for early diagnosis.

Interestingly, in an experimental gingivitis model, asymptomatic and suboptimal gum health was associated with a shift in plaque microbiome structure, plaque metabolome, and host immune response during gingivitis onset and progression [30].

This metabolomics study was conducted on whole saliva from a healthy young population. Using the FMBS as a classification parameter, we investigated whether a particular saliva composition could reflect the early stages of gingival inflammation (preclinical stage of gingivitis). We speculated that subjects with high FMBS scores but still within the normal range might have a different composition of salivary metabolites than subjects with low FMBS scores. According to our metabolomics analysis, detecting a signature of specific salivary metabolites could help identify individuals who may be more susceptible to gingival inflammation based on their FMBS scores.

## 2. Results

### 2.1. Clinical Data of the Study Participants

The enrolled subjects’ demographic data, dental/periodontal indexes, and whole saliva flow are shown in Tables S1 and S2. All the participants had a normal salivary function, with salivary flow ranging from 0.9 to 5 mL/5 min. Their FMBS scores ranged from 0 to 11.4%, and their Full-Mouth Plaque Score (FMPS) ranged from 2.8 to 24.8% (Table S2). Only one of the twenty participants had an FMBS score higher than 10% (11.4%), which is the highest value compatible with a gingivitis-free state [13]; this finding resulted in the exclusion of this subject from subsequent evaluations. The observed FMPS scores indicated adequate plaque control, and Periodontal Screening and Recording (PSR) indices excluded the presence of active periodontal disease. Aggregate data of the selected subjects (n = 19) are presented in Table 1.

### 2.2. Eukaryotic and Prokaryotic Cell Counts in Saliva

Procaryotic and eukaryotic cell counts performed in whole saliva samples showed a relevant variability compared to their mean values (Table S3). No significant correlations were found between the FMBS scores and the number of prokaryotic or eukaryotic cells suspended in whole saliva (R = 0.43 with *p*-value = 0.07 and R = 0.40 with *p*-value = 0.09, respectively) and between the FMPS scores and the number of prokaryotic or eukaryotic cells (R = 0.19 with *p*-value = 0.43 and R = 0.003 with *p*-value = 0.99, respectively).

### 2.3. Bleeding Score and Salivary Metabolomes Analysis

**Figure 1:**
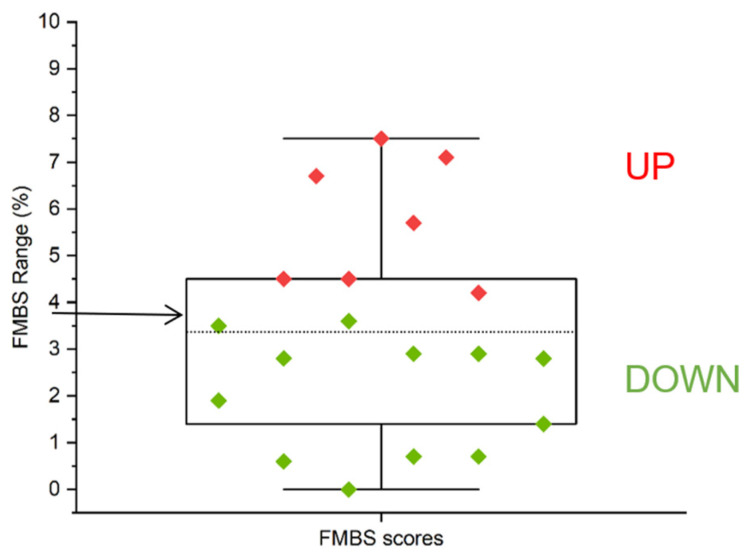
FMBS score distribution. The arrow at y = 3.75% corresponds to the FMBS threshold selected for the enrolled subjects’ arbitrary UP and DOWN categorization (see Section 4). Green diamonds have FMBS values < 3.75% (DOWN group), and red diamonds have FMBS values > 3.75% (UP group). The boxes are determined by the 25th and 75th percentiles; the dotted line corresponds to the mean (3.4%).

We selected an FMBS of 3.75%, the midpoint of the observed FMBS range (0–7.5%), as a suitable threshold for investigating the metabolite composition of saliva samples to search for metabolite alterations associated with the preclinical stage of gingivitis. According to our classification based on FMBS score, 12 subjects were allocated to the group with FMBS < 3.75% (DOWN) and 7 to the group with FMBS > 3.75% (UP) (Figure 1).

**Figure 2:**
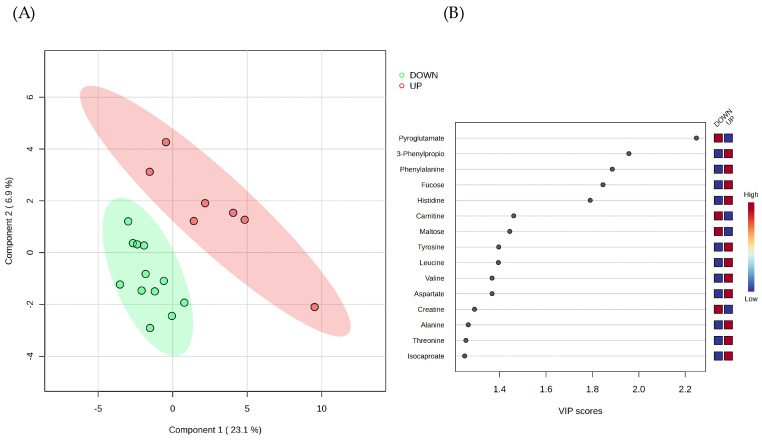
Supervised multivariate analysis of the salivary metabolite datasets. (**A**) Partial Least Squares–Discriminant Analysis (PLS-DA) scores plot of whole saliva metabolomes. The classification model separates the DOWN (FMBS < 3.75%) from the UP (FMBS > 3.75%) salivary metabolomes. Components 1 and 2 account for 23.1% and 6.9% of the variance, respectively, and colored ellipses represent each cluster’s 95% confidence region. (**B**) Metabolite ranking (top 15 metabolites) according to the Variable’s Importance in Projection (VIP) scores, resulting from PLS-DA component 1. The higher the VIP score of a variable, the better its ability to discriminate between groups. Variables with a VIP score close to or greater than 1 are considered relevant [31]. The blue and red boxes on the right denote the relative metabolite abundance in the two clusters.

We applied Partial Least Squares–Discriminant Analysis (PLS-DA) to the concentrations of the sixty metabolites identified in the whole saliva samples of the two groups of subjects. The obtained PLS-DA model was effective in separating the metabolomes of the UP and DOWN groups (Figure 2A), and the Variable’s Importance in Projection (VIP) score plot suggested the metabolites with the highest contribution to the separation by component 1 (Figure 2B).

Among the top 15 metabolite features identified by the VIP score plot, we arbitrarily selected a VIP score ≥ 1.8 to extract the variables that contribute most to class discrimination in the PLS-DA model. These variables are Pyroglutamate, 3-Phenylpropionate, Phenylalanine, Fucose, and Histidine (Figure 2B).

**Figure 3:**
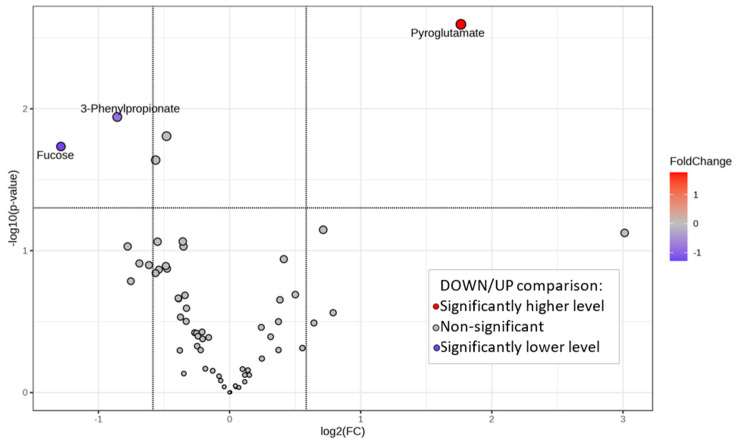
Volcano plot of whole saliva metabolomes shows the statistically significant metabolites. Fold change (FC) threshold of 1.5 and a *p*-value < 0.05 were considered for significance. Significantly different metabolite concentrations are highlighted.

The volcano plot in Figure 3 highlights the significantly different metabolite concentrations observed in the UP and DOWN groups.

**Figure 4:**
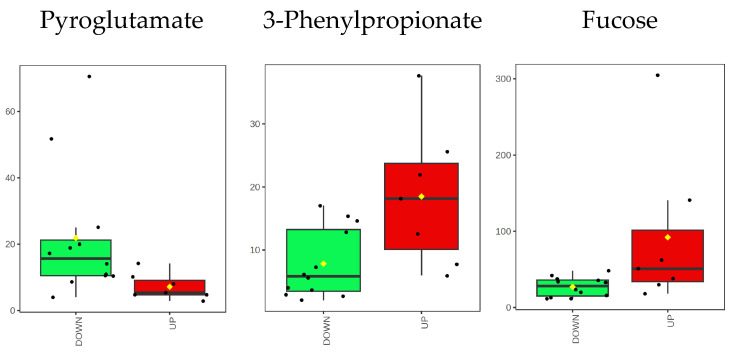
Box plots of the distributions of the concentration (μM) of the discriminant metabolites. Black dots represent the metabolite concentrations. The black line inside each boxplot is the median, and the yellow diamond is the mean.

When comparing the DOWN vs. UP group, Pyroglutamate shows a higher concentration, while 3-Phenylpropionate and Fucose show a lower concentration. A graphical summary of their concentration distributions is displayed in Figure 4.

Figure S1 provides the DOWN vs. UP comparison of all metabolite concentrations to complement the statistical analysis above.

### 2.4. Biomarker Evaluation by Receiver Operating Characteristic (ROC) Curve Analysis

Classical univariate ROC curve analysis was conducted using all variables as potential classification elements to identify the metabolites that perform better in predicting UP or DOWN group membership. The analysis identified six metabolites with area under the ROC curve (AUC) scores greater than 0.82 and statistical significance for the *t*-test with a *p*-value < 0.05 (Table 2, Figure S2). This response indicates that the single concentrations of Pyroglutamate, Maltose, Histidine, Fucose, Phenylalanine, and 3-Phenylpropionate have reasonable accuracy in discriminating between the UP and DOWN groups. However, their AUC values primarily indicate the biomarker potential of the single variables rather than their predictive performance.

**Figure 5:**
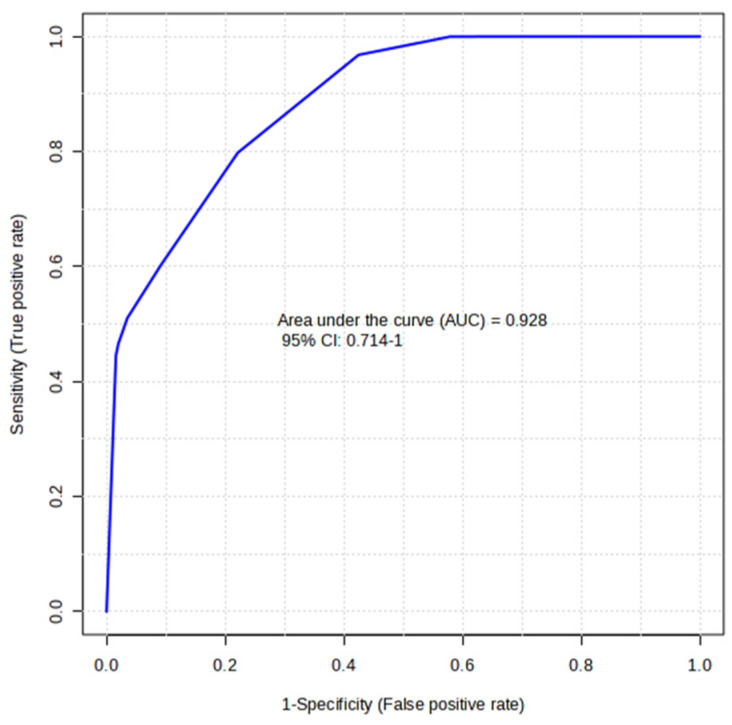
Multivariate ROC curve as a predictive model based on selected salivary metabolites. The ROC curve was constructed using Pyroglutamate, Fucose, and 3-Phenylpropionate normalized concentrations.

In multivariate analysis, the group-discriminating ability of selected salivary metabolites was used to develop a predictive model. We chose the combination of the three metabolites Pyroglutamate, Fucose, and 3-Phenylpropionate differentially expressed in the DOWN and UP groups (Figure 3) to generate an ROC curve-based model (Figure 5). With an AUC of 0.93, the analysis attributed a good discriminatory power to the metabolite ensemble. The average accuracy based on cross-validations was 0.74. However, the predicted group probability of each sample, resulting from 100 cross-validations, classified three of the DOWN samples in the wrong group, indicating that they might be outliers.

In a subsequent analysis aimed at predicting the category (DOWN vs. UP) of new samples, the obtained model was tested on the whole saliva metabolome of the subject excluded from our study because his FMBS was higher than 10%. The metabolite profile of this subject fitted into the DOWN group, albeit with an FMBS of 11.4%.

## 3. Discussion

The current gold standard for diagnosing periodontal diseases is based on clinical examination with a periodontal probe, which may be combined with a radiographic examination. However, clinical and radiographic assessments provide a picture of what has happened to the patient in the past. Still, they cannot predict how the disease will progress. Instead, the identification of biomarkers has the potential to detect the disease at an early stage, before clinical signs appear, and forecast disease progression or response to treatment.

As end-products of many physiological and pathological processes, metabolites are markers of specific pathways that also result from host-microbial interactions, such as gingival inflammation [32]. Metabolites originating from gingival tissues and plaque bacteria are likely released in the crevicular fluid and eventually in saliva [33]. The ability to detect specific salivary signatures of early gingival inflammation, even at a preclinical level, may be paramount to identifying individuals liable to developing periodontal disease not intercepted by current clinical parameters.

We previously detailed the metabolic composition of whole, parotid, and submandibular/sublingual saliva of the healthy subjects involved in this study, finding a certain degree of individual variability in the metabolite composition of salivary samples [19,24]. In the present study, we arbitrarily selected the FMBS score of 3.75% as a threshold to classify the study participants based on their tendency to gingival bleeding. This threshold has enabled the selection of subjects with FMBS scores near or above the 75th percentile of the distribution (UP group, Figure 1) to compare their salivary metabolomes with those with a minor tendency for gingival bleeding (DOWN group). We expect the UP group to be more prone to gingival inflammation. PLS-DA analysis separated the saliva samples into two clusters according to the bleeding score threshold (Figure 2A). Further statistical analysis revealed that Pyroglutamate, 3-Phenylpropionate, and Fucose concentrations differ significantly between participants with FMBS> and <3.75% (Figure 3). As expected, they are on the list of metabolites with the highest contribution to cluster separation in the PLS-DA model (Figure 2B).

Pyroglutamate is found in whole saliva due to the cyclization reaction of Glutamine or Glutamic acid. This reaction occurs when these residues are at the N-terminus of several human salivary proteins, such as in salivary α-amylase [34]. According to our analysis, high levels of pyroglutamate characterize the salivary composition of subjects with low FMBS scores in the DOWN group (Figure 2B, Figure 3 and Figure 4).

3-Phenylpropionate is a metabolic product of aromatic amino acid fermentation by anaerobic bacteria of the subgingival plaque [35]. We detected 3-Phenylpropionate only in whole saliva, reflecting its microbial metabolic origin [19]. Subjects with FMBS scores > 3.75% exhibited higher levels of 3-Phenylpropionate (Figure 2B, Figure 3 and Figure 4), suggesting the contribution of oral microflora to this alteration. They also had higher levels of Phenylalanine, Histidine, and other amino acids (UP group in Figure 2B). Salivary Phenylalanine concentration has been reported to correlate with the proteolytic bacterial load, indicating that oral bacteria can generate that metabolite [36]. Interestingly, bacterial or endogenous proteases’ degradation of salivary proteins is considered the primary source of salivary free amino acids also in plaque-induced gingivitis [37].

Fucose has a higher salivary concentration among subjects of the UP group, Figure 2B, Figure 3 and Figure 4. Such a finding is supported by the study of Shetty and Pattabiraman [38], in which they demonstrated that Fucose, determined as a protein-bound fraction, is higher in patients with gingivitis and periodontitis than in healthy subjects. Wsoo and Ahmed showed that salivary total Fucose and fucose-related parameters were significantly increased in patients with advanced and moderate periodontitis compared to healthy subjects [39]. Roopa et al. observed an increase in salivary Fucose levels in periodontitis, probably caused by a rise in fucosidase activity associated with the breakdown of plasma and tissue glycoproteins caused by inflammation [40]. We hypothesize that the higher concentration of salivary Fucose in subjects with FMBS > 3.75% might reflect an increase in oral bacteria fucosidase activity, functional to the production of a free form of the carbohydrate [24].

Overall, our analysis has demonstrated that (a) the UP group (FMBS score > 3.75) is associated with higher salivary levels of specific amino acids (Phenylalanine, Histidine, Tyrosine, Leucine, Valine, Aspartate, Alanine, and Threonine, Figure 2B) and Fucose and lower levels of Pyroglutamate, and (b) the alterations in salivary metabolites that can distinguish between the groups with FMBS> or <3.75% may be attributed to oral bacteria metabolism. Remarkably, oral microflora perturbances have been recognized as involved in major clinical conditions, such as gingivitis and periodontitis [41].

It is worth noting that neither the FMBS nor FMPS scores of the study participants were correlated with eukaryotic or prokaryotic cell counts obtained from whole saliva samples. This could be because we only counted planktonic cells. It is important to acknowledge that oral microorganisms are organized with specificity in different oral niches, such as saliva, tooth, and soft tissue surfaces, and those that contribute most to gingival inflammation and bleeding are expected to adhere to teeth and gingiva as plaque biofilm [42].

Univariate ROC analysis was used to explain the performance of individual metabolites in predicting UP or DOWN group membership. Pyroglutamate, Maltose, Histidine, Fucose, Phenylalanine, and 3-Phenylpropionate were found to produce ROC curves with an AUC ranging from 0.99 to 0.82, thereby demonstrating a reasonable ability to discriminate between the two groups (Table 2, Figure S2). This suggests that the salivary levels of these metabolites could potentially serve as indicators of group membership.

By selecting the combination of Pyroglutamate, 3-Phenylpropionate, and Fucose variables, of which the concentrations were significantly different between the subjects with FMBS> or <3.75%, multivariate ROC analysis generated a model with an AUC of 0.93, indicating an acceptable predictive accuracy. When this ROC-based model was tested on the metabolite dataset of a subject with an FMBS score of 11.4%, the sample was assigned to the DOWN group despite the FMBS score. A possible explanation for this result is that the sample was obtained from a subject with localized gingivitis. This condition may have caused metabolite alterations that differ from those of healthy subjects with an FMBS score > 3.75% but still within the normal range.

We acknowledge that an FMBS within the range of 3.75–10% does not indicate the presence of gingivitis or any periodontal alteration and that our results are conditioned by the original study design and sample size, which were not specifically intended for the current purpose. As the study has a cross-sectional design, it did not provide information on any developments over time in subjects with higher FMBS scores. A longitudinal approach is needed to gain information on the possible development of gingivitis in at-risk subjects.

To confirm the validity of our biomarker model, a more comprehensive study with a larger cohort of subjects is required. The inclusion of two additional groups of patients with localized and generalized gingivitis (FMBS 10–30% and FMBS > 30%, respectively) will contribute to the validation of our metabolomics approach.

The complex pathogenesis of periodontal disease suggests that the golden key to diagnosing the onset of gingivitis should be based on a combination of factors rather than metabolites alone. Our biomarker model should be integrated with the analysis of molecules involved in innate and adaptive oral immunity, such as cytokines. These could not be targeted in the current analysis because the NMR samples, according to our sample preparation procedure [43], have been protein-depleted.

In clinical practice, the diagnosis of periodontal diseases can only assess the advanced stage of the disease, not its onset or evolution. These pathologies are not linear and are characterized by periods of progression and remission [44,45]. A salivary metabolite signature could allow early-stage periodontal diagnosis, offering an easy, safe, and non-invasive approach to planning appropriate treatments. Transferring scientific findings to clinical practice is also relevant for developing rapid, low-cost, and accurate point-of-care technologies, thus improving the personalized approach to precision medicine [46].

## 4. Materials and Methods

### 4.1. Ethics Statement

The protocol of this pilot study was approved by the Ethics Committee of “Area Vasta Emilia Nord” (AVEN) (protocol number: 808/2018/SPER/UNIPR METASAL3). The study was conducted according to the criteria set by the Declaration of Helsinki. Written informed consent was obtained from all the eligible subjects before enrolment in the study.

### 4.2. Clinical Assessment of the Study Participants

The cohort of volunteers enrolled in this study was previously included in the project aimed at metabolite profiling of different saliva subtypes (whole, parotid, and submandibular/sublingual saliva) and serum [19,24].

Participant selection and enrolment were conducted at the Centro Universitario di Odontoiatria of the University of Parma, Italy, and lasted three months (March–May 2019). Inclusion and exclusion criteria for participant selection have been previously reported [24]. Twenty healthy subjects (10 males and 10 females) aged between 20 and 25 years were consecutively recruited. During selection, eligible subjects underwent a comprehensive oral exam and whole sialometry (by modified Saxon Test) [47]. Subjects with hyposalivation (whole saliva flow < 1 mL/5 min), systemic or oral diseases affecting dental or periodontal tissues, medication-induced salivary dysfunction, and pregnant or lactating women were excluded from the study. Any history of successfully treated periodontitis was an exclusion criterion.

A single trained dental specialist performed a thorough oral examination with the help of a mirror and periodontal probe (UNC15, University of North Carolina), including teeth, periodontal tissues, and oral mucosa (alveolar, labial, buccal mucosa, and mucosa covering tongue, palate, and attached gingiva). In doubtful cases, intraoral radiography and/or orthopantomography were performed to detect dental lesions that visual examination could not identify.

Dental status was assessed using the Decayed, Missing and Filled Teeth (DFMT) index. Periodontal health was assessed using the Periodontal Screening and Recording (PSR) index, Full-Mouth Plaque Score (FMPS), and Full-Mouth Bleeding Score (FMBS) [48]. FMPS and FMBS scores were assessed at six sites per tooth.

We used restrictive/precautionary measures for smoke, drugs, and alcohol confounders. For the subjects identified as ‘light smokers’ in Table S1, we required at least a 12 h smoking restriction before collecting saliva. We also verified that salivation was unaffected in the three drug-using participants listed in Table S1. Eight subjects were identified as “moderate drinkers”, consuming less than 7 alcohol units per week (Table S1). Selected subjects were asked to refrain from eating and strenuous exercise for at least 12 h before the saliva sample was taken and to drink only water. They were also asked to avoid oral hygiene (brushing and flossing) for 45 min before saliva collection. Immediately before saliva collection, patients rinsed their mouths with water for 1 min. Unstimulated whole saliva collection was performed using the passive drooling method between 8:00 am and 10:00 am to minimize the influence of the circadian rhythm on salivary composition [24].

### 4.3. Cell Counting in Whole Saliva

Eukaryotic and prokaryotic cells were counted in whole saliva samples, according to Gardner et al. [49]. Eukaryotic cells (mainly oral epithelial cells and leucocytes) were counted on the same day of saliva collection. Briefly, 20 μL of each untreated salivary sample were mixed with 20 μL of 0.4% Trypan blue (Sigma-Aldrich, Poole, Dorset, UK), placed in a hemocytometer counting chamber, and counted with a light microscope Nikon eclipse TS100 (Nikon, Tokio, Japan) (100× magnification).

For prokaryotic cells, 2 μL of thawed saliva samples were heat-fixed to a glass slide and Gram-stained [50]. Cells were counted with a Nikon Eclipse 80i microscope using at least four random fields at 100× magnification. Each count was performed by two independent observers using at least four random fields. In all cases, variability was less than 10%.

### 4.4. NMR Metabolomics

NMR sample preparation was performed according to an optimized protocol for NMR-based metabolomics [43]. Briefly, saliva samples were assembled as follows: 10 μL of 1 M potassium phosphate buffer (pH 7.4) and 15 μL of 1% 3-trimethylsilylpropionic acid (TSP) in D_2_O were added to 575 μL of the filtered saliva (achieving final concentrations of 1.45 mM TSP and 2.5% D_2_O in 16 mM phosphate buffer). TSP was used as a reference for chemical shift (0.00 ppm) and quantitative internal standard.

One-dimensional ^1^H-NMR spectra were acquired at 25 °C with a JEOL 600 MHz ECZ600R spectrometer using the first increment of the 1DNOESY pulse sequence, 128 scans, a sweep window of 20 ppm, 128 k points, and a relaxation delay of 5 s. The spectra were processed by zero-filling to 256 k points and line broadening at 0.5 Hz and analyzed. Metabolite identification and quantification were performed using Chenomx NMR suite 8.3 software (Chenomx Inc., Edmonton, AB, Canada) [51]. The chemometric analysis allowed the identification and quantification of sixty metabolites in all saliva samples, resulting in a whole saliva metabolic profile described in detail elsewhere [19].

Statistical analysis: Descriptive statistics summarized the subjects’ data using mean ± SD (Origin 2019 software). Spearman correlation coefficient was computed to evaluate the relationship between FMBS or FMPS and the number of cells (procaryotic or eucaryotic) suspended in whole saliva samples; the significance level was set at *p* < 0.05.

Metabolomics data analysis: We preliminary classified the population into two groups based on the observed FMBS scores. The midpoint of the observed FMBS range (3.75%, [0–7.5%]), which approximately marks the boundary between the scores above and below the 75th percentile, was chosen as the threshold for assigning subjects to the UP (>3.75%) or DOWN (<3.75%) group (Figure 1).

Statistical analysis on metabolite datasets was carried out using the MetaboAnalyst 6.0 platform (www.metaboanalyst.ca, accessed on 1 March 2024). Metabolite concentration data were uploaded according to the group membership (UP or DOWN), normalized by the median value to adjust for systematic differences between samples, and auto-scaled (mean-centered and divided by the standard deviation of each metabolite concentration) to adjust for fold differences between variables. We applied supervised Partial Least Squares Discriminant Analysis (PLS-DA) and obtained the Variable’s Importance in Projection (VIP) score for the variables that contributed most to group separation. We generated a volcano plot by combining the results from FC Analysis and t-test (FC threshold of 1.5 and a *p*-value < 0.05 for significance).

Potential biomarkers for classification in the UP or DOWN group were predicted by classical univariate Receiver Operating Characteristic (ROC) curve analysis. The area under the curve (AUC) was used to compare the performance of different variables. Multivariate ROC curve analysis was applied based on selected variables to generate a predictive model for group classification. The multivariate ROC curves were based on the cross-validation performance of the Support Vector Machine (SVM) classification method. To produce a smooth ROC curve, 100 cross-validations were performed, and the results were averaged to generate the final curve plot.

## 5. Conclusions

To the best of our knowledge, our pilot study is the first to assess the metabolite composition of whole saliva from healthy subjects according to their FMBS scores. We identified a panel of metabolites differentially expressed in healthy subjects with high but physiological FMBS scores compared to those with lower scores. Due to the study’s limited sample size, our results must be considered exploratory. However, as this set of metabolites may be associated with enzymatic activities of oral bacteria, it may help to identify individuals more susceptible to gingival inflammation. By expanding the study population to include patients with different degrees of gingival bleeding, we are confident that salivary metabolomics will help identify suboptimal conditions of gingival health and contribute to future point-of-care diagnostics.

## Figures and Tables

**Table 1:** Demographics, oral health status, and social habits of the selected subjects.

	MALE (n = 10)	FEMALE (n = 9)
	Mean ± SD	Mean ± SD
Age (years)	23.7 ± 1.3	23.6 ± 2.1
BMI (kg/m^2^)	23.2 ± 1.4	20.9 ± 1.3
Salivary flow ^a^ (mL/5 min)	2.3 ± 1.2	2.2 ± 1.5
% FMPS	12.8 ± 7.1	14.1 ± 7.5
% FMBS	2.6 ± 1.9	4.6 ± 3.4
DMFT	1.7 ± 1.3	1.0 ± 1.3
PSR	0.9 ± 0.3	1 ± 0.0
	No. of subjects	No. of subjects
Drugs: - In the last 12 h - No drugs		
	-	3 ^b^
	10	6
Smoke: - Cigarette smokers ^c^ - Non-smokers		
	2	3
	8	6
Alcohol: - Moderate drinkers ^d^ - Non-drinkers		
	4	4
	6	5

^a^ Determined by modified Saxon test; ^b^ contraceptive (2 subjects) or antihistamine therapy (1 subject); ^c^ up to 5 cigarettes/day; ^d^ less than 7 alcohol units/week.

**Table 2:** Significant metabolites in univariate ROC curve analysis.

Metabolite	AUC ^§^	Optimal Cutoff *	*p*-Value
Pyroglutamate	0.99	−0.66	0.0025
Maltose	0.90	−0.45	0.0749
Histidine	0.88	−0.24	0.0229
Fucose	0.88	−0.14	0.0184
Phenylalanine	0.83	−0.23	0.0156
3-Phenylpropionate	0.82	0.30	0.0114

^§^ AUC, area under the Receiver Operating Characteristic (ROC) curve; * optimal cutoff is the metabolite normalized concentration with the best performance in discriminating the DOWN and UP groups.

## Data Availability

Data is contained within the article.

## Supplementary Materials

The following supporting information can be downloaded at https://www.mdpi.com/article/10.3390/ijms25105448/s1.
