# Supplementary material for: The Association between Salivary Metabolites and Gingival Bleeding Score in Healthy Subjects: A Pilot Study
Source: Int J Mol Sci. 2024 May 17;25(10):5448. doi: 10.3390/ijms25105448 (PMC11122368; doi:10.3390/ijms25105448)
Supplement: Supplementary file 1 [file ijms-25-05448-s001.zip › ijms-2987435-supplementary.pdf]

Association between salivary metabolites and gingival bleeding score in healthy subjects. A pilot study

Rita Antonelli[1*], Elena Ferrari[2*], Mariana Gallo[2], Tecla Ciociola[3], Elena Calciolari[1,4], Alberto Spisni[2#], Marco Meleti[1], Thelma A. Pertinhez[2]

[1] Centro Universitario di Odontoiatria, Department of Medicine and Surgery, University of Parma, Parma, Italy

[2] Laboratory of Biochemistry and Metabolomics, Department of Medicine and Surgery, University of Parma, Parma, Italy

[3] Laboratory of Microbiology and Virology, Department of Medicine and Surgery, University of Parma, Parma, Italy

[4] Center for Oral Clinical Research, Institute of Dentistry, Barts and The London School of Medicine and Dentistry, Queen Mary University of London, London UK

*These two authors contributed equally to this work (Elena Ferrari: co-first author)

#Corresponding author: Alberto Spisni, Department of Medicine and Surgery, University of Parma, Parma, Italy. E-mail: alberto.spisni@unipr.it

SUPPLEMENTARY MATERIAL

Contents:

Table S1. Demographic data of the study participants

Table S2. Dental/periodontal parameters and whole saliva flow of the study participants

Table S3. Eukaryotic and prokaryotic cell counts obtained from whole saliva samples

Figure S1. Differences in metabolite concentrations between DOWN and UP groups.

Figure S2. Univariate ROC curve analysis for selected metabolite features.

Table S1. Demographic data of the study participants

Subject	Sex	Age (years)	Height (cm), weight (Kg)	Drugs in the last 12h	Smoke
01	Male	25	176, 79	/	Occasionally
02	Female	25	160, 57	/	/
03	Male	24	175, 68	/	/
04	Male	23	187, 83	/	/
05	Female	25	167, 70	/	/
06	Male	25	175, 68	/	/
07	Female	25	170, 62	/	e-cigarette
08	Female	24	163, 50	Birth-control pill	/
09	Male	25	183, 80	/	/
10	Female	25	170, 65	/	/
11	Male	21	175, 72	/	/
12	Female	24	164, 52	/	Yes (5/die)
13	Female	25	170, 62	Birth-control pill	/
14	Male	23	170, 72	/	/
15	Male	23	170, 63	/	/
16	Male	23	180, 75	/	Occasionally
17	Female	20	177, 64	/	/
18	Female	24	170, 58	Antihistamine	/
19	Female	20	165, 60	/	Yes (5/die)
20	Male	25	175, 65	/	/

Table S2. Dental/periodontal parameters and whole saliva flow of the study participants

Subject	DMFT	PSR	FMBS (%)	FMPS (%)	Whole Saliva Flow (mL/5 min)
01	0	1	5.7%	12.1%	3.0
02	0	1	7.5%	18.4%	1.1
03	1	1	2.9%	16.8%	2.3
04	3	1	0.7%	9.3%	2.0
05	0	1	11.4%	5.0%	2.4
06	2	0	0%	4.3%	1.3
07	0	1	0.7%	8.3%	5.0
08	0	1	3.6%	5.7%	1.9
09	1	1	0.6%	6.8%	2.2
10	5	1	2.9%	19.6%	1.1
11	4	1	2.8%	2.8%	1.0
12	4	1	2.8%	7.7%	4.5
13	2	1	4.5%	24.8%	2.4
14	2	1	3.5%	16.4%	5.0
15	2	1	4.2%	22.4%	2.1
16	3	1	4.5%	23.4%	1.2
17	0	1	1.4%	9.9%	1.8
18	0	1	6.7%	18.8%	1.1
19	2	1	7.1%	22.7%	0.9
20	0	1	1.9%	14.1%	3.0

DFMT: *Decayed, Missing, and Filled Teeth*; PSR: *Periodontal Screening and Recording;* FMBS: *Full Mouth Bleeding Score,* FMPS: *Full Mouth Plaque Score*

Table S3. Eukaryotic and prokaryotic cell counts obtained from whole saliva samples

Subject	Prokaryotic cells (cell/mL)	Eukaryotic cells (cells/mL)
01	1.95E+09	7.75E+05
02	3.71E+09	5.10E+05
03	1.10E+09	1.35E+05
04	2.52E+09	1.50E+04
05	N.d.	7.80E+05
06	1.06E+09	2.85E+05
07	1.16E+09	3.00E+04
08	1.17E+09	2.09E+06
09	2.63E+08	2.40E+05
10	1.11E+09	7.75E+05
11	5.46E+08	5.75E+05
12	9.89E+08	4.40E+05
13	1.02E+08	7.50E+04
14	4.05E+08	8.00E+04
15	7.00E+08	4.70E+05
16	2.85E+09	1.45E+06
17	1.51E+09	4.60E+05
18	3.39E+09	4.95E+05
19	2.08E+09	2.05E+05
20	1.71E+08	3.90E+05
Mean	***1.41E+09***	***5.14E+05***
St dev	***1.07E+09***	***5.04E+05***

N.d. not determined

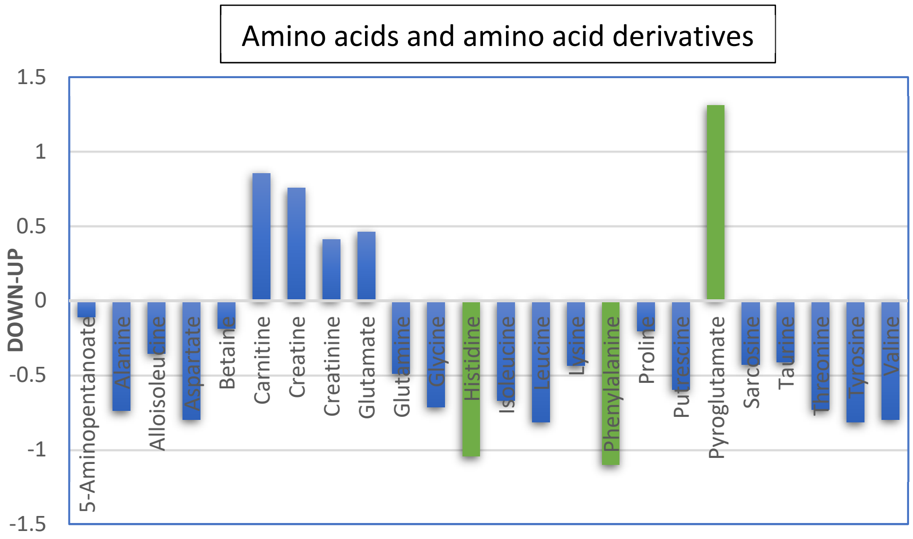
Amino acids and amino acid derivatives
DOWN-UP
1.5
1
0.5
0
-0.5
-1
-1.5
5-Aminopentanoate
Alanine
Alloisoleucine
Aspartate
Betaine
Carnitine
Creatine
Creatinine
Glutamate
Glutamine
Glycine
Histidine
Isoleucine
Leucine
Lysine
Phenylalanine
Proline
Putrescine
Pyroglutamate
Sarcosine
Taurine
Threonine
Tyrosine
Valine

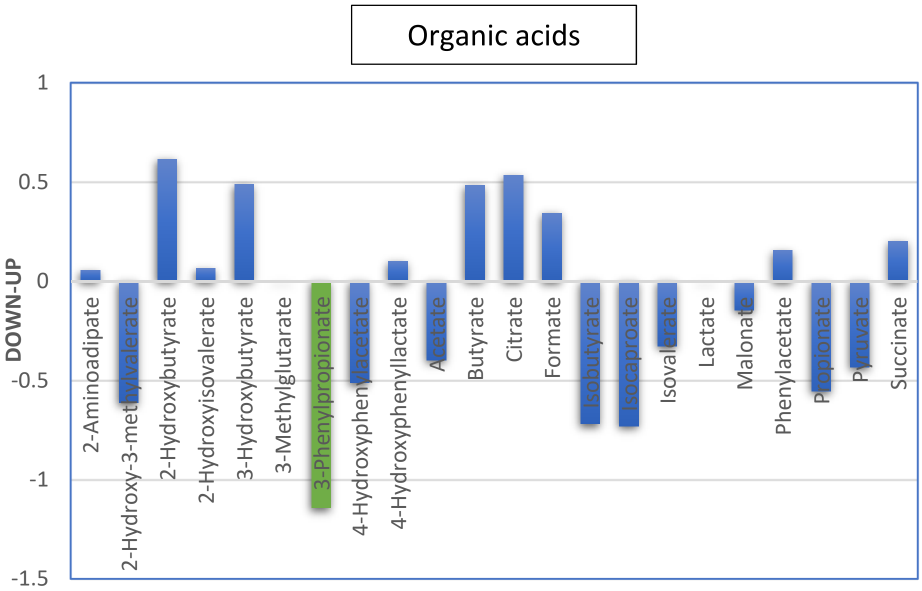
Organic acids
DOWN-UP
1
0.5
0
-0.5
-1
-1.5
2-Aminoadipate
2-Hydroxy-3-methylvalerate
2-Hydroxybutyrate
2-Hydroxyisovalerate
3-Hydroxybutyrate
3-Methylglutarate
3-Phenylpropionate
4-Hydroxyphenylacetate
4-Hydroxyphenyllactate
Acetate
Butyrate
Citrate
Formate
Isobutyrate
Isocaproate
Isovalerate
Lactate
Malonate
Phenylacetate
Propionate
Pyruvate
Succinate

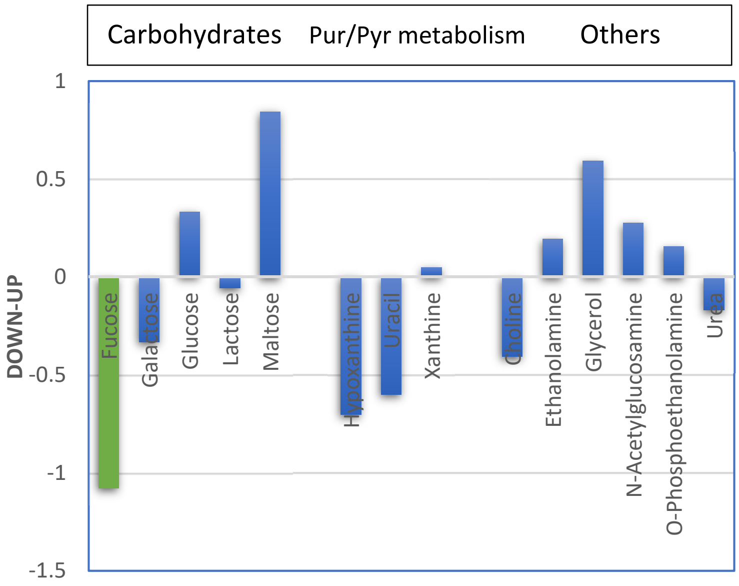
Carbohydrates
Pur/Pyr metabolism
Others
DOWN-UP
1
0.5
0
-0.5
-1
-1.5
Fucose
Galactose
Glucose
Lactose
Maltose
Hypoxanthine
Uracil
Xanthine
Choline
Ethanolamine
Glycerol
N-Acetylglucosamine
O-Phosphoethanolamine
Urea

Figure S1. Differences in metabolite concentrations between DOWN and UP groups. Metabolite concentrations have been normalized by the median and autoscaled. The graph displays the difference between the mean values obtained for the UP and DOWN groups (DOWN-UP) for all salivary metabolites. The green bars correspond to the metabolites with a DOWN-UP difference higher than ±1, which coincides with the top five VIP metabolites identified by PLS-DA analysis.

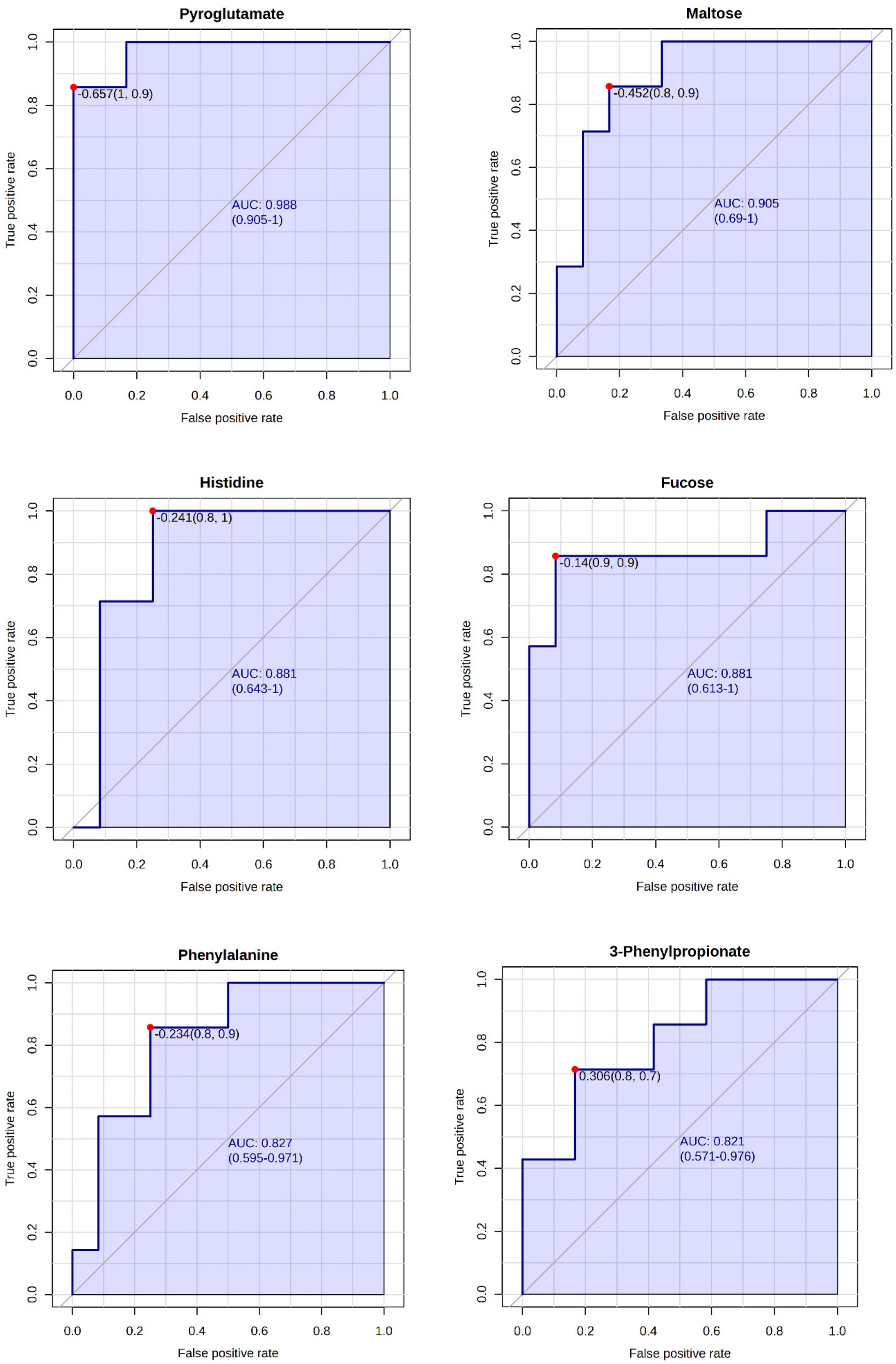

Figure S2. Univariate ROC curve analysis for selected metabolite features. The solid red dot indicates the optimal cutoff with the associated sensitivity and specificity values.
